# Development and Validation of a Combined Ferroptosis and Immune Prognostic Model for Melanoma

**DOI:** 10.1155/2022/1840361

**Published:** 2022-11-24

**Authors:** Mingsui Tang, Yaling Li, Fang Wang, Jiande Han, Yali Gao

**Affiliations:** ^1^Department of Dermatology, The First Hospital of China Medical University, Shenyang 110001, Liaoning, China; ^2^Center for Translational Medicine Research and Development, Shen Zhen Institutes of Advanced Technology, Chinese Academy of Science, Shenzhen 518055, Guangdong, China; ^3^Department of Dermatology, The First Affiliated Hospital, Sun Yat-Sen University, Guangzhou 510080, Guangdong, China

## Abstract

**Background:**

Melanoma development and progression are significantly influenced by ferroptosis and the immune microenvironment. However, there are no reliable biomarkers for melanoma prognosis prediction based on ferroptosis and immunological response.

**Methods:**

Ferroptosis-related genes (FRGs) were retrieved from the FerrDb website. Immune-related genes (IRGs) were collected in the ImmPort dataset. The TCGA (The Cancer Genome Atlas) and GSE65904 datasets both contained prognostic FRGs and IRGs. The model was created using multivariate Cox regression, the least absolute shrinkage and selection operator (LASSO) Cox regression analysis, and the analysis and comparison between the expression patterns of ferroptosis and immune cell infiltration were done. Last but not least, research was conducted to assess the expression and involvement of the genes in the comprehensive index of ferroptosis and immune (CIFI).

**Results:**

Two prognostic ferroptosis- and immune-related markers (*PDGFRB* and *FOXM1*) were utilized to develop a CIFI. In various datasets and patient subgroups, CIFI exhibits consistent predictive performance. The fact that CIFI is an independent prognostic factor for melanoma patients was revealed. Patients in the CIFI-high group further exhibited immune-suppressive characteristics and had elevated ferroptosis gene expression levels. The results of in vitro research point to the possibility that the *PDGFRB* and *FOXM1* genes function as oncogenes in melanoma.

**Conclusion:**

In this study, a novel prognostic classifier for melanoma patients was developed and validated using ferroptosis and immune expression profiles.

## 1. Introduction

Melanoma is the deadliest type of skin cancer, with a yearly rise in incidence [1, 2]. The limited treatment of choice for advanced melanoma is immune checkpoint blockade (ICB) and molecularly targeted therapies, such as CTLA-4, PD-1/PD-L1 inhibitors, and BRAF inhibitors due to melanoma's high level of heterogeneity and aggressiveness [3, 4]. However, primary or secondary drug resistance affects more than 50% of melanoma patients, which presents a significant clinical treatment challenge [5, 6]. In addition to the timing of treatment being delayed once patients develop drug resistance, there will be limited options for subsequent therapeutic approaches [7]. Developing precise, individualized treatment plans for various patients and accurately predicting the risk and drug treatment response for different individuals is an effective approach to addressing the drug resistance problem. Obtaining reliable biomarkers for a melanoma diagnosis is therefore crucial to initiate clinical treatment. Ferroptosis is an excessive lipid peroxidation-induced regulated cell death mode that is iron-dependent and associated with the progression and treatment response of multiple types of tumors [8, 9]. Recent research has demonstrated that ferroptosis may result in immunosuppression brought by inflammation in the tumor microenvironment [10, 11]. Additionally, there are interactions between immune and tumor cells that are connected to ferroptosis [12, 13]. Ferroptosis is regulated by a variety of molecular components in the tumor microenvironment. Increased iron accumulation, free radical generation, fatty acid supply, and lipid peroxidation are critical for the development of ferroptosis [14]. Numerous studies have demonstrated that the immunosuppressive microenvironment can be impacted by ferroptosis intervention. Ferroptosis may expose tumor antigens, increasing the tumor microenvironment's immunogenicity and enhancing the effectiveness of immunotherapy [15–17].However, the JAK-STAT1 pathway is activated, and SLC7A11 and SLC3A2 expression are downregulated, which causes ferroptosis in tumor cells when interferon gamma is released by cytotoxic T cells [18]. The long-term effects of ferroptosis on tumor immunity depend on the interactions among cancer cells and other immune cell subsets. For instance, the lymphatic system inhibits melanoma cells from ferroptosis via the mechanism of enhancing the synthesis of ACSL3-dependent MUFAs, which promote tumor spread [19]. Despite a strong link between ferroptosis effects and the immune microenvironment, their role in melanoma is yet unknown. In this work, a comprehensive index of ferroptosis and immune (CIFI) model was created and validated using ferroptosis-related genes (FRGs) and immune-related genes (IRGs). The CIFI model demonstrated consistent prognostic predictive performance in patients with various clinical features and across various datasets. The outcomes of in vitro experiments and clinical validation were used to validate the expression and function of the *PDGFRB* gene and the *FOXM1* gene in CIFI.

## 2. Materials and Methods

### 2.1. Data Preparation

RNA-seq information and follow-up data for melanoma patients were acquired from TCGA (472 samples). Data from the Gene Expression Omnibus (GEO) were obtained, and the GSE65904 dataset (214 samples) was chosen since it had the biggest sample set in the GEO database and detailed follow-up information. The FerrDb website includes FRGs. The ImmPort dataset's IRGs were downloaded.

### 2.2. Construction and Validation of the CIFI

We selected the independent prognostic genes across FRGs and IRGs using the “survival” program and univariate Cox analysis. Both LASSO analysis and sequential Cox proportional hazards regression were employed to develop CIFI. The risk score's optimal cutoff value was used to establish the CIFI-high and CIFI-low groups (categories). To examine the differences (variations) in overall survival between the CIFI-high and CIFI-low groups, the Kaplan-Meier survival analysis was utilized. The time-dependent ROC analysis was also used to assess CIFI's predictive ability. To assess the CIFI's independent prognostic significance, both univariate and multivariate Cox regression analyses were utilized.

### 2.3. Potentially Regulatory Pathways Analysis

The score of each pathway per sample was determined using a single-sample gene set enrichment analysis (ssGSEA) with the aid of the “GSVA” package. The analysis of the correlation between the CIFI and ssGSEA scores of each sample was then conducted using the potential regulatory pathways.

### 2.4. Immunohistochemistry (IHC) Analysis

The First Hospital of China Medical University provided clinical samples (paired nontumor skin tissue and melanoma). According to a prior study [20], IHC staining and scoring were conducted. The following antibodies were used: *PDGFRB* (1 : 200; ab69506; Abcam) and *FOXM1* (1 : 1000; ab207298; Abcam). The Ethics Committee of the First Hospital of China Medical University approved this study.

### 2.5. Cell Culture and Transfection

All of the cell lines (PIG1, A375, A875, and MeWo) used in this investigation were provided by the China Infrastructure of Cell Line Resource. PIG1 is immortalized dermal melanocytes, A375 is human malignant melanoma cells, A875 is human melanoma cell, and MeWo is human malignant melanoma cells. Small interfering RNA (siRNA) and Lipofectamine 2000 (Invitrogen, Shanghai, China), as previously described [20], were used for cell transfection. *PDGFRB*-siRNA had the following sequences: 5′-GGAAUGAGGUGGUCAACUU-3′. Additionally, the *FOXM1*-siRNA sequences were 5′-GGACCACUUUCCCUACUUU-3′.

### 2.6. CCK8 Assay and Colony-Forming Experiments

The negative control siRNA (NC-siRNA), the *PDGFRB*-specific siRNA, and the *FOXM1*-specific siRNA were all transfected into cell cultures in 96-well plates. Cells were cultured with CCK8 solution (C0038, Beyotime, Shanghai, China) for another 2 hours after 0, 24, 48, and 72 hours. To determine cell vitality, an optical density (OD) value at 450 nm was recorded. Cells (500/well) treated with siRNAs for colony-forming experiments were added to 12-well plates. The colonies were counted after two weeks.

### 2.7. qPCR and Western Blot

qPCR and western blot procedures were conducted as previously mentioned [21].

### 2.8. Statistical Analysis

The statistical tool SPSS 21.0 was used to examine the data (IBM Corporation, Armonk, NY, USA). Software called GraphPad Prism 8.0 was used to generate the graphs (GraphPad Software, Inc., San Diego, CA). Student's *t*-tests were applied. With regard to *t*-tests, a two-tailed *p* < 0.05 indicated a significant value.

## 3. Results

### 3.1. Development of CIFI in Melanoma

**Figure 1:**
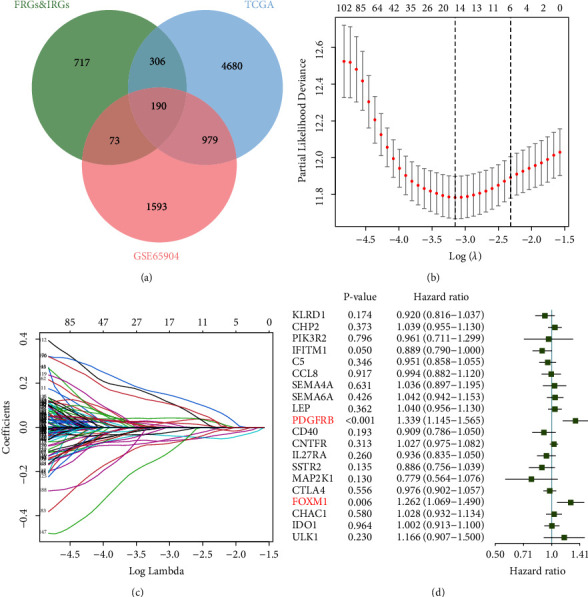
Prospective FRGs and IRGs identified in melanoma. (a) 190 predicted FRGs and IRGs were detected in the GSE65904 and TCGA cohorts, using a Venn diagram. (b) 100-foldcross-validation for LASSO model-related parameter selection refinement. (c) The most significant prognostic genes' LASSO coefficient profiles. (d) *PDGFRB* and *FOXM1* show statistical significance in the Cox proportional hazards regression model incorporating 20 genes.

Using univariate Cox regression analysis, prognostic genes were initially discovered using the TCGA dataset and the GSE65904 dataset. In total, 4680 prognostic genes involved from the TCGA dataset were filtered out. In Supplement Figure 1A, the top 20 genes' hazard ratios (ranked by *P* value) were displayed. In addition, 1593 prognostic genes were screened from the GSE65904 dataset, and Supplement Figure 1B showed the top 20 genes' hazard ratios (ordered by *P* value). In addition, 190 prognostic FRGs and IRGs were the intersecting genes between the GSE65904 and TCGA cohorts (Figure 1(a)). The optimal prognostic genes were then chosen using the LASSO Cox regression model, resulting in a model incorporating 20 genes: *KLRD1, CHP2, PIK3R2, IFITM1, C5, CCL8, SEMA4A, SEMA6A, LEP, PDGFRB, CD40, CNTFR, IL27RA, SSTR2, MAP2K1, CTLA4, FOXM1, CHAC1, IDO1*, and *ULK1* (Figures 1(b) and 1(c)). This model was examined and optimized using a stepwise Cox proportional hazards model to include the optimal prognostic genes, resulting in a final set of two genes (Figure 1(d)). Consequently, CIFI was developed: CIFI = (0.292 × *PDGFRB* expression) + (0.233 × *FOXM1* expression).

### 3.2. TCGA Dataset Prognostic Analysis of CIFI

**Figure 2:**
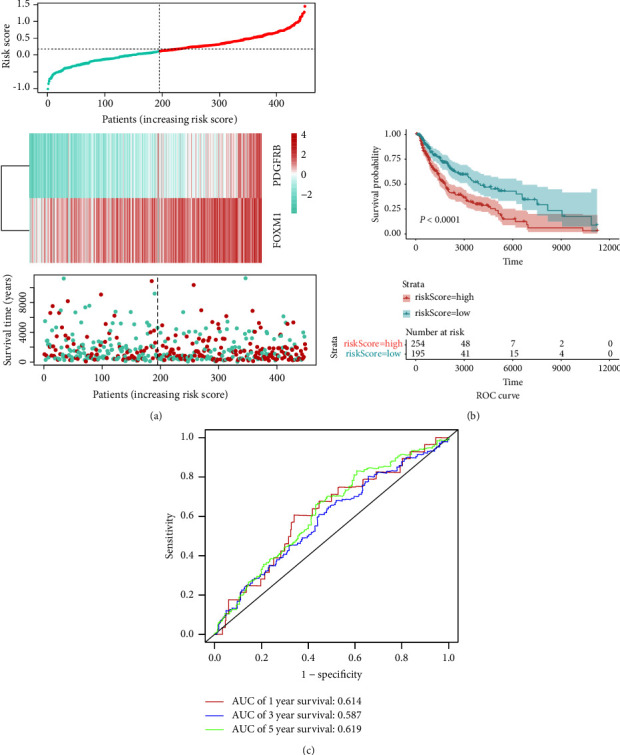
CIFI-related prognostic analysis in the TCGA dataset. (a) Risk scores survival duration, survival status, and *PDGFRB* and *FOXM1* expression levels in CIFI. (b) Kaplan-Meier-based comparison of the OS between the groups with high and low CIFI. (c) Time-dependent ROC analysis of CIFI for OS and survival status.

The risk score distribution from the TCGA dataset, which was initially computed for each sample in Figure 2(a), was shown. Patients in the CIFI-high group showed remarkably worse overall survival rates than those in the CIFI-low group, as per a Kaplan-Meier survival analysis (Figure 2(b); *P* < 0.0001). The AUC values over 1, 3, and 5 years of survival were shown by ROC curve analysis to be 0.614, 0.587, and 0.619, respectively (Figure 2(c)).

### 3.3. Verification of CIFI in GSE65904

**Figure 3:**
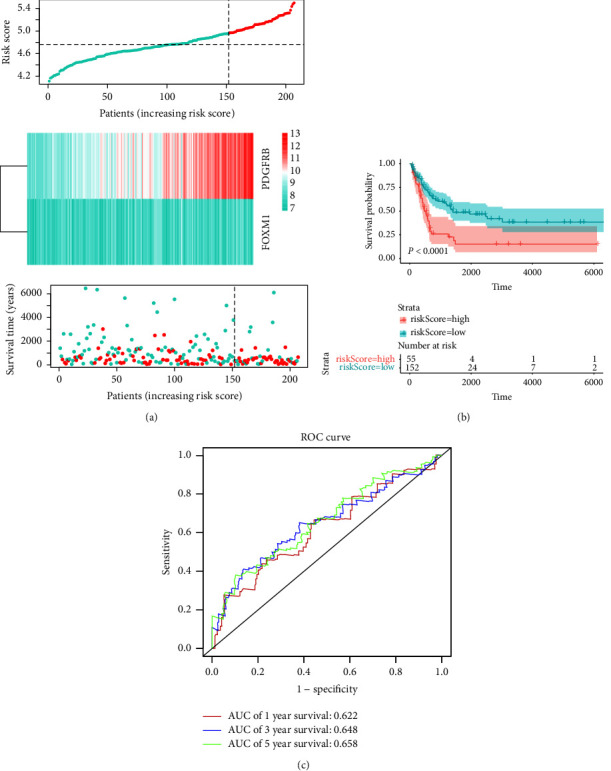
Validation of CIFI in the GSE65904 dataset. (a) Risk scores, survival times, survival status, and expressions of *PDGFRB* and *FOXM1* in CIFI. (b) Kaplan-Meier analysis of the OS in the CIFI-high group versus the CIFI-low group. (c) Time-dependent ROC analysis of CIFI for OS and survival status.

The stability and dependability of CIFI are then further verified.  Figure 3(a) displayed the GSE65904's CIFI distribution. The overall survival of patients in the CIFI-high group was significantly lower than that of patients in the CIFI-low group, as per a Kaplan-Meier prognostic analysis in Figure 3(b) (*P* < 0.0001). AUC values of 0.622, 0.648, and 0.658 were found for 1-year, 3-year, and 5-year survival, respectively, based on ROC curve analysis (Figure 3(c)).

### 3.4. Prognostic Value of CIFI in Various Melanoma Patient Subgroups

**Figure 4:**
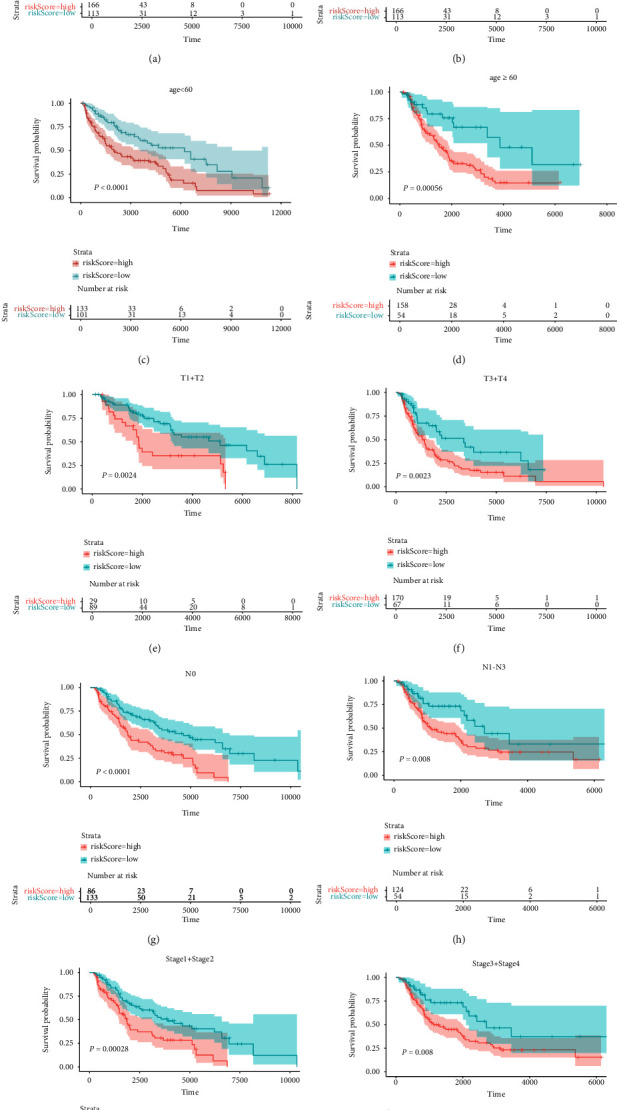
Impact of CIFI on prognosis in melanoma patients with distinct clinical characteristics. (a) Male. (b) Female. (c) Age <60. (d) Age ≥60. (e) *T*1 + *T*2. (f) *T*3 + *T*4. (g) *N*0. (h) *N*1–*N*3. (i) Stage 1 + Stage 2. (j) Stage 3 + Stage 4.

Next, individuals with distinct clinical features were studied to determine the prognostic significance of CIFI. Figures 4(a)–4(j) showed that among melanoma patients with different clinical characteristics, the overall survival of patients belonging to the CIFI-high group was considerably worse than that of the CIFI-low group. However, CIFI was able to differentiate the prognosis of several patient subgroups.

### 3.5. Cox Analysis of CIFI and Nomogram Construction

**Figure 5:**
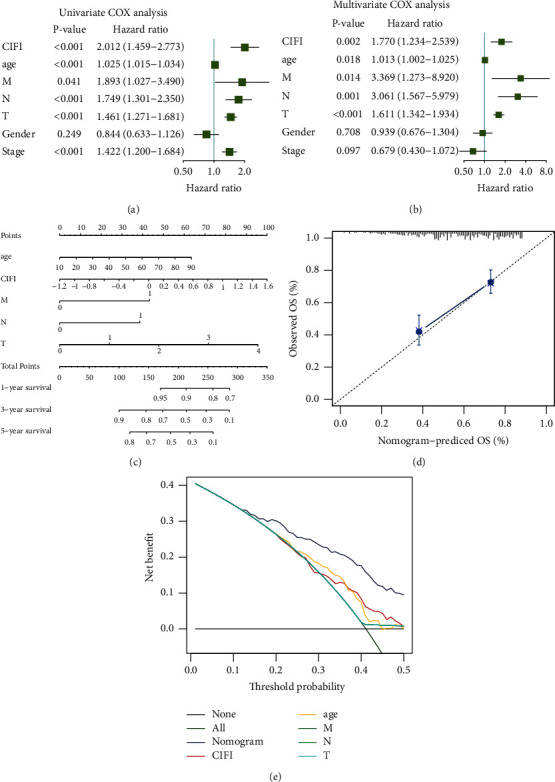
CIFI Cox analysis and nomogram model development. (a) Univariate Cox analysis of OS in the TCGA dataset. (b) Multivariate Cox analysis of OS in the TCGA dataset. (c) A nomogram model that incorporates CIFI and conventional clinical characteristics. (d) The nomogram model's 5-year calibration curves. (e) DCA (decision curve analysis) of the nomogram model.

We then performed univariate and multivariate Cox regression analyses. Age, CIFI, M, N, T, and tumor stage were all linked to patients' prognosis, according to a univariate Cox analysis (Figure 5(a)). According to a multivariate Cox analysis, the patient's prognosis was determined independently by CIFI, age, *M* stage, *N* stage, and *T* stage (Figure 5(b)). These findings showed that a high CIFI was an independent predictor of outcomes in melanoma patients.

The development of a quantitative technique in clinical practice may also aid doctors in assessing melanoma patients' prognosis. Based on the outcomes of multivariate Cox regression analysis, a nomogram incorporating clinicopathological traits and CIFI was constructed (Figure 5(c)). The model's potent ability to predict patient outcomes over 5 years was confirmed by the calibration curves, which revealed considerable overlap between the calibration points and the standard curve (Figure 5(d)). Additionally, the decision curve analysis demonstrated that the nomogram model accurately predicted overall survival (OS) compared to a single clinicopathological characteristic (Figure 5(e)). These findings revealed the therapeutic use of the CIFI-based nomogram to determine the prognosis of melanoma patients.

### 3.6. Immune Profile in the CIFI

**Figure 6:**
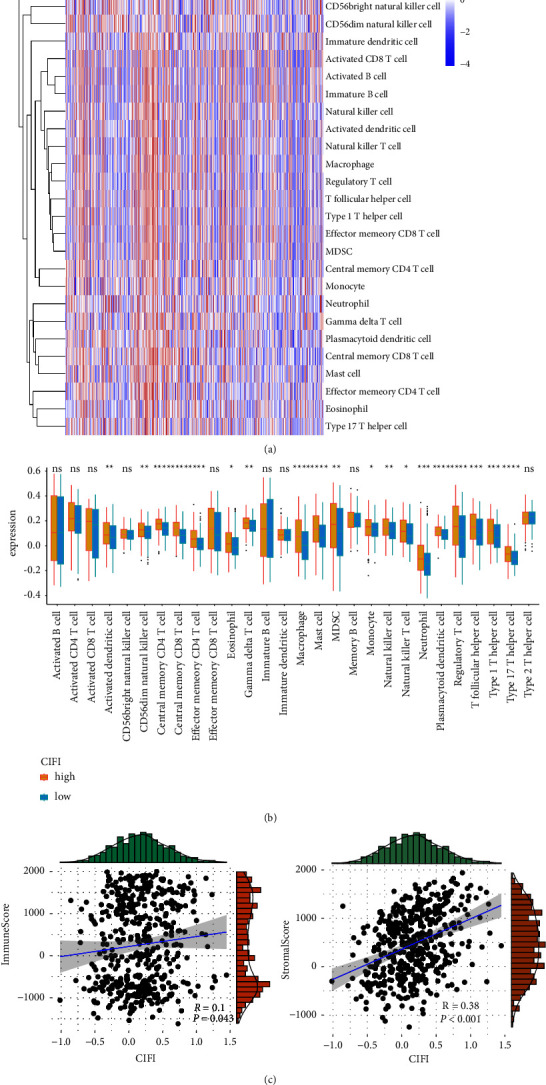
CIFI's immune profile. (a, b) Comparison of the distribution of 28 different kinds of immune cells between the CIFI-high and CIFI-low groups. (c) Correlations between CIFI and immune score, and correlations between CIFI and stromal score.

We then investigated if CIFI may indicate a melanoma immunological state. First, we investigated the link between CIFI and immune-invading cells. ssGSEA was conducted to determine the level of infiltration of 28 immune cells. Some immune cells were expressed aberrantly in both the CIFI-high and CIFI-low groups (Figures 6(a) and 6(b)). Following that, we sought to investigate the link between CIFI and the tumor immune microenvironment.  Figure 6(c) showed that CIFI was positively correlated with immune score (*R* = 0.1, *P* < 0.043) and stromal score (*R* = 0.38, *P* < 0.001).

### 3.7. Identifying Pathways Related to CIFI

**Figure 7:**
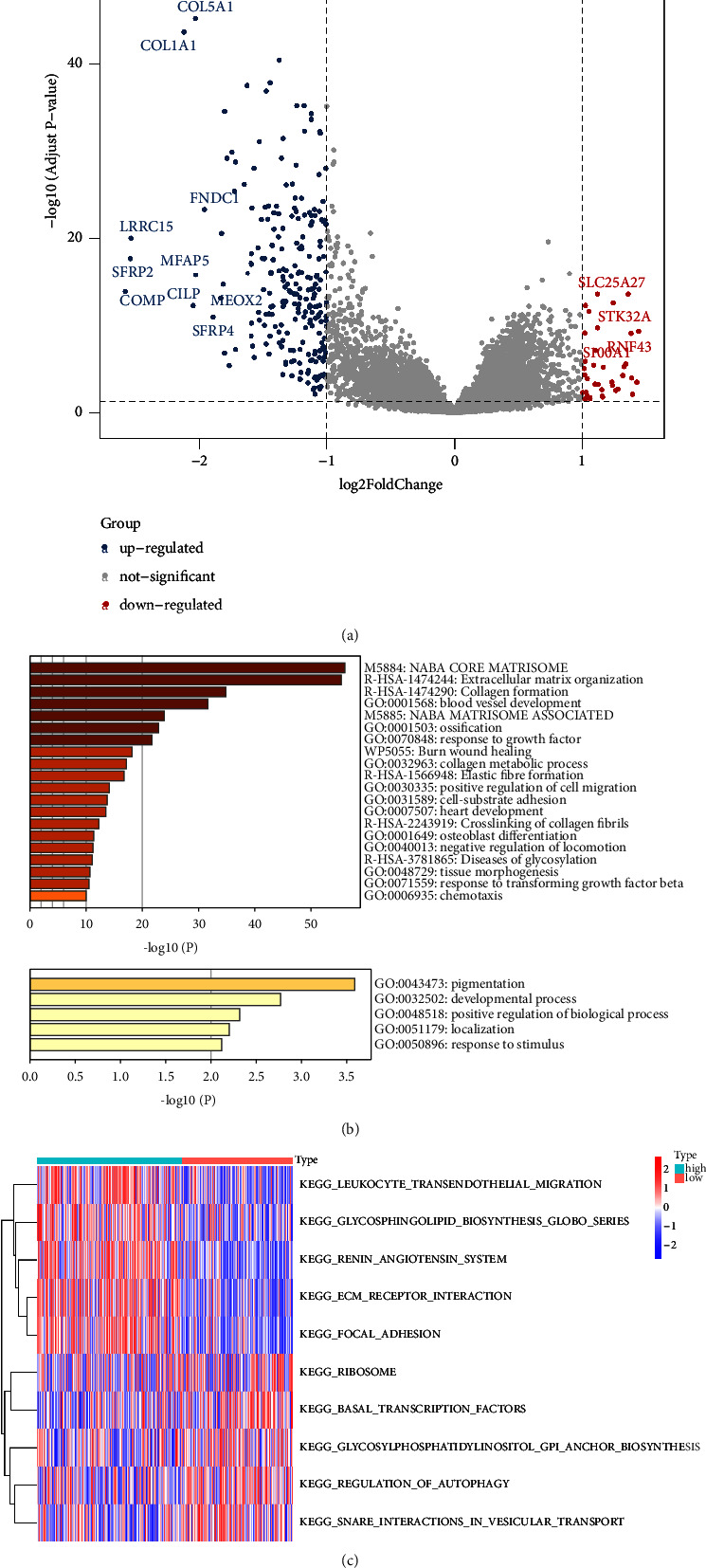
Identifying pathways related to CIFI. (a) The volcano plot compares DEGs between the CIFI-high and CIFI-low groups in the TCGA cohort. (b) Based on remarkably upregulated and downregulated genes, respectively, a gene ontology enrichment analysis was carried out. (c) Based on GSVA analysis, the heatmap depicts the various expressed pathways in the CIFI-high and CIFI-low groups.

The link between CIFI and biological function was the subject of our next analysis effort. Using a cutoff of *P* < 0.05, it was observed that the TCGA cohort samples with CIFI-high had 237 substantially upregulated genes and 38 considerably downregulated genes (Figure 7(a)). The expression levels of these differentially expressed genes (DEGs) were then imported into Metascape. The extracellular matrix organization, collagen synthesis, blood vessel development, etc. were the primary areas where the upregulated genes were enriched. The majority of the downregulated genes were specialized for pigmentation, inner ear development, etc. (Figure 7(b)). The scores of all patients in various routes were then obtained by calculating the ssGSEA scores by GSVA. As the risk score grew, ECM receptor interaction, focal adhesion, and other activities increased, whereas ribosome, basal transcription factors, and other activities decreased (Figure 7(c)).

### 3.8. Validation of the CIFI Genes at the mRNA and Protein Levels, and Its Functional Analysis

**Figure 8:**
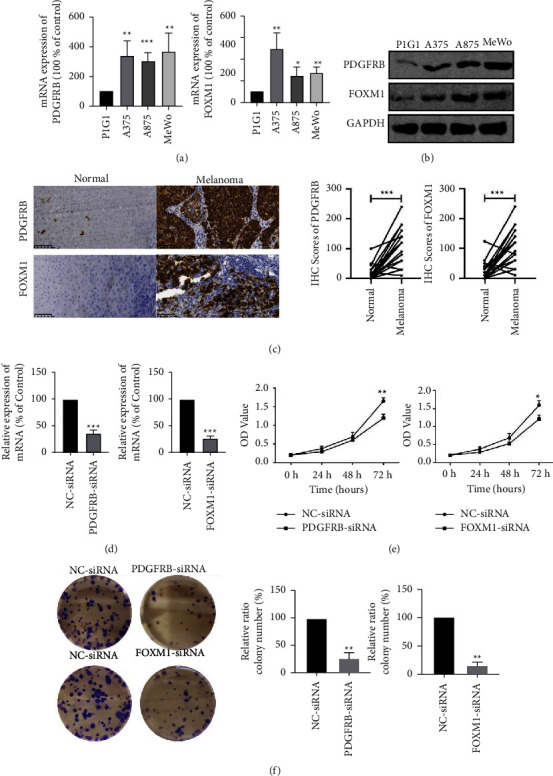
mRNA and protein level validation of PDGFRB and FOXM1 and the functional analysis in melanoma against normal samples. (a, b) The mRNA and protein expression of two genes based on melanocytes (PIG1) and melanoma cells (A375, A875, and MeWo). (c) IHC examination of PDGFRB and FOXM1 in healthy and malignant tissue. (d) Transfection efficiency of A375 cells. (e) CCK8 assay (A375). (f) Colony formation assays (A375). Data represent mean ±SD (standard deviation); ^∗^*P* < 0.05, ^∗∗^*P* < 0.01 and ^∗∗^*P* < 0.001 (versus control group).

As shown in Figures 8(a) and 8(b), melanoma cells (A375, A875, and MeWo) had considerably higher *PDGFRB* and *FOXM1* mRNA and protein levels than melanocyte PIG1 cells. The outcomes of immunohistochemistry experiments demonstrated that *PDGFRB* and *FOXM1* were overexpressed in melanoma samples in comparison to normal tissues (Figure 8(c)). Furthermore, we transfected the A375 cells, and transfection efficiency was shown in Figure 8(d). Based on in vitro experiments, we found that silencing *PDGFRB* and *FOXM1* inhibited the proliferative capacity of melanoma cells (Figures 8(e) and 8(f)). These findings collectively indicated that *PDGFRB* and *FOXM1* may act as carcinogens in melanoma.

## 4. Discussion

Melanoma is highly aggressive, yet the patient's prognosis remains poor due to limited conventional treatment options [22, 23]. Therefore, there is a need to establish prognostic characteristics for melanoma patients. Several studies have provided potential prognostic assessment models for melanoma patients [24–26]. However, most of the studies were genomic or transcriptomic-based and did not validate biological functions. Melanoma onset and progression are significantly influenced by ferroptosis and the immune microenvironment [27, 28]. We developed CIFI in this study utilizing FRGs and IRGs based on open-source datasets. In many datasets and patient subsets, CIFI exhibits consistent predictive accuracy. Significantly, CIFI is an independent prognostic factor for melanoma patients. In conclusion, CIFI has the potential to be useful in clinical settings and is quite effective in predicting the prognosis of patients with melanoma.

Currently, several studies have reported immune-related molecular markers as prognostic markers for melanoma [29–31], but these prognostic markers had limitations. First, molecular markers in previous studies contained various genes, which increased the workload and cost in clinical practice and constrained the clinical utility of these molecular markers to some extent [29, 32, 33]. Second, these studies have not further investigated the underlying mechanisms or clinical significance of prognostic markers [28, 34], and therefore, the clinical reliability of these prognostic markers was unclarified. Therefore, it is imperative to find a reliable and practical prognostic marker. Our study yields a predictive model for melanoma patients based on ferroptosis and immunity, two essential tumor characteristics. This model may simultaneously reflect changes in melanoma's ferroptosis and immunological state. Our methodology is also practical for clinical use. Most importantly, the outcomes of clinical samples and in vitro tests have validated the expression level and probable functions of genes in our model. The CIFI we constructed contains two genes, *PDGFRB* and *FOXM1*. Among these, *PDGFRB* and *FOXM1* are genes involved in ferroptosis and the immune system, respectively. Embryonic development, cell proliferation, survival, differentiation, chemotaxis, and migration are all regulated by the protein that the *PDGFRB* gene produces [35, 36]. Previous studies have established the link between *PDGFRB* overexpression and a poor prognosis in patients with renal cell carcinoma [37], oral squamous cell carcinoma [38], ovarian cancer [39], and colorectal cancer [40]. Involved in cell proliferation, the transcriptional activator *FOXM1* may have an impact on the expression of several cell cycle genes, including cyclin B1 and cyclin D. Numerous studies have revealed the association of elevated *FOXM1* expression levels with a poor prognosis in patients with ovarian cancer [41], pancreatic and esophageal cancers [42], malignant rhabdoid tumors [41], and small-cell lung cancer [43]. *FOXM1* expression level is elevated and activated in malignant melanoma [44]. *FOXM1* inhibition might be a promising treatment strategy for metastatic melanoma [45].

We also compared our model with others. Only two models based on immune or ferroptosis-related genes have been discovered so far [46, 47]. However, in our analysis, for the first time, the incorporated ferroptosis and immune gene set were utilized to develop a melanoma-related prognostic model, which could play a consistent prognostic performance in diverse data sets that could be utilized as an independent prognosis-related predictive indicator for melanoma patients.

Although CIFI has the potential to be an excellent model for predicting prognosis in individuals with melanoma, it has certain drawbacks. To begin, all samples in this study were obtained retrospectively, and potential samples are currently being validated. We, therefore, examined CIFI's prognostic significance in clinic settings. The roles of *PDGFRB* and *FOXM1* in CIFI require further in vivo and in vitro investigations.

## 5. Conclusions

In conclusion, a novel prognostic classifier based on ferroptosis and immune expression profiles in patients with melanoma was developed and validated.

## Data Availability

The data that support the findings of this study are available from the corresponding author upon reasonable request.

## Supplementary Materials

Supplementary Materials Supplement Figure 1: the hazard ratio of the top 20 genes (sorted by *P*-value) in (A) TCGA dataset and (B) GSE65904 dataset.
