# Supplementary figures and images for: Development and Validation of a Combined Ferroptosis and Immune Prognostic Model for Melanoma
Source: J Oncol. 2022 Nov 24;2022:1840361. doi: 10.1155/2022/1840361 (PMC9715341; doi:10.1155/2022/1840361)

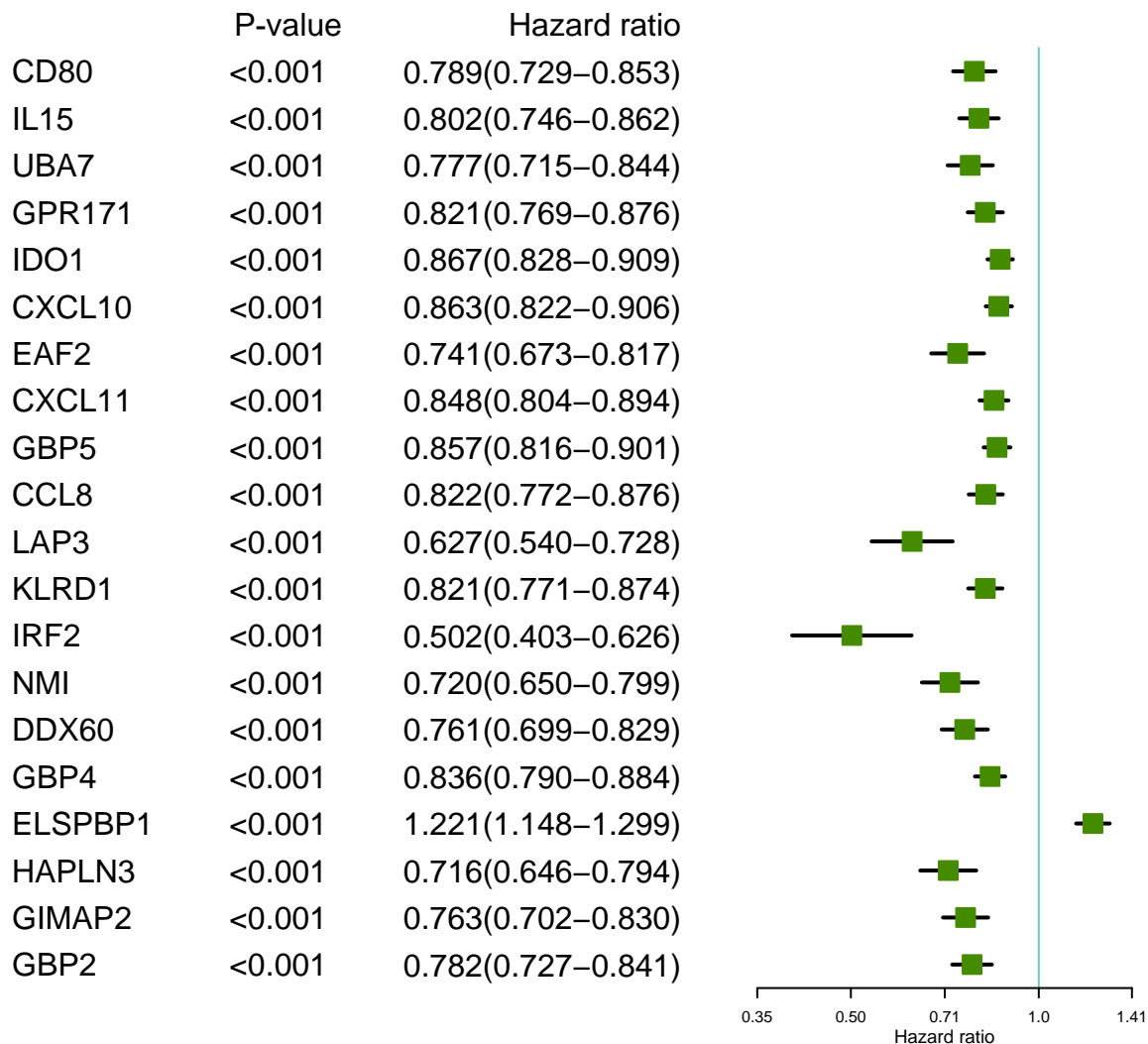

Supplement: Supplementary Materials — Supplement Figure 1: the hazard ratio of the top 20 genes (sorted by P-value) in (A) TCGA dataset and (B) GSE65904 dataset. [file 1840361.f1.zip › Supplement Figure 1A.pdf]

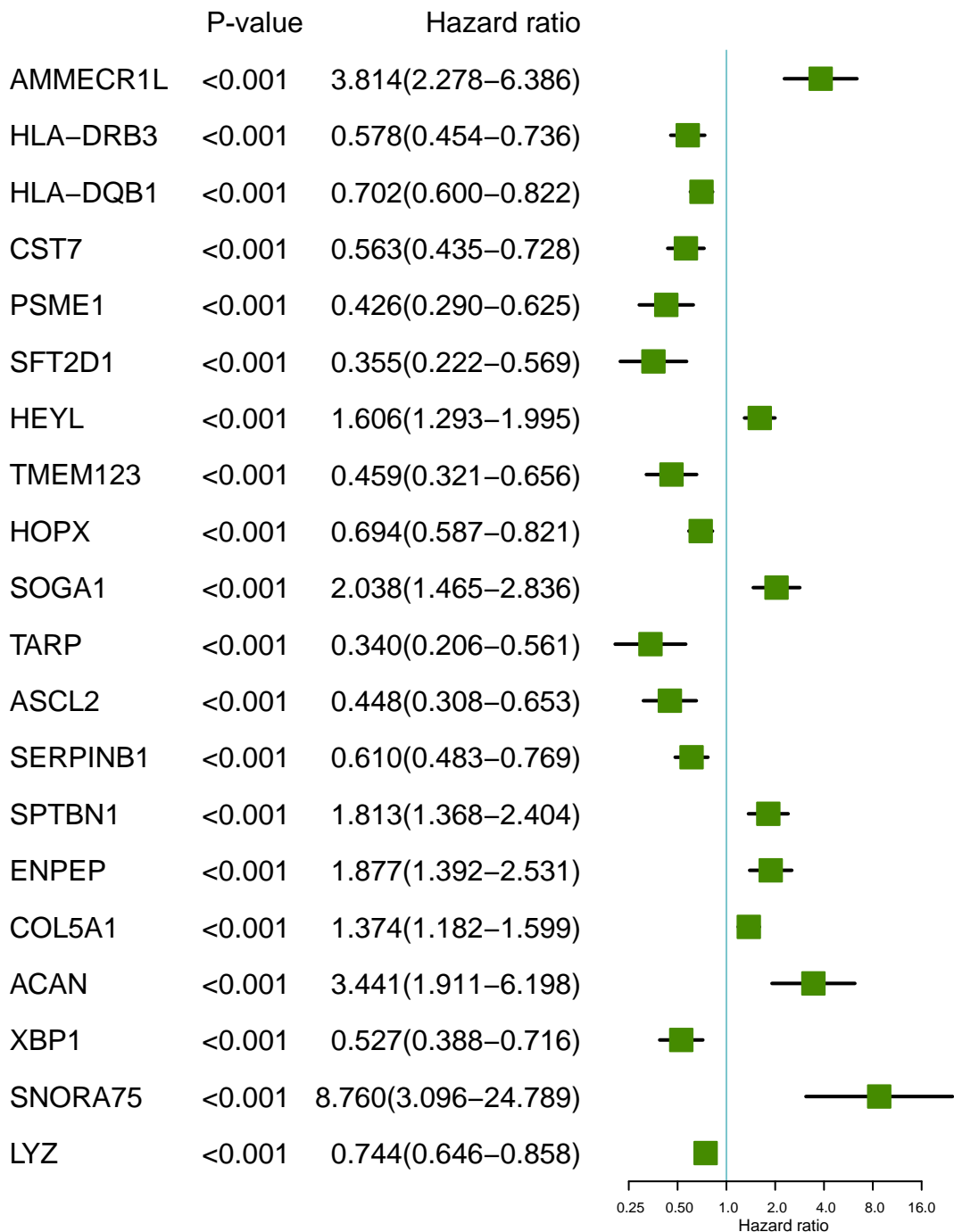

Supplement: Supplementary Materials — Supplement Figure 1: the hazard ratio of the top 20 genes (sorted by P-value) in (A) TCGA dataset and (B) GSE65904 dataset. [file 1840361.f1.zip › Supplement Figure 1B.pdf]
